# What do the implementation outcome variables tell us about the scaling-up of the antiretroviral treatment adherence clubs in South Africa? A document review

**DOI:** 10.1186/s12961-019-0428-z

**Published:** 2019-03-14

**Authors:** Ferdinand C. Mukumbang, Zaida Orth, Brian van Wyk

**Affiliations:** School of Public Health, University of the Western Cape, Cape Town, South Africa

**Keywords:** Adherence clubs, implementation research, antiretroviral treatment, adherence, retention in care, document review, South Africa

## Abstract

**Background:**

The successful initiation of people living with HIV on antiretroviral treatment (ART) in South Africa fomented challenges of poor retention in care and suboptimal adherence to medication. Following evidence of the potential of adherence clubs (ACs) to improve patient retention in ART and adherence to medication, the South African National Department of Health drafted a policy in 2016 encouraging the rollout of ACs nationwide. However, little guidance on the rollout strategy has been provided to date, and the national adoption status of the AC programme is unclear. To this end, we aimed to review the effectiveness of the rollout of the antiretroviral AC intervention in South Africa to date through an implementation research framework.

**Methods:**

We utilised a deductive thematic analysis of documents of the AC programme in South Africa obtained from searching various databases from December 2017 to July 2018. The implementation outcome variables (acceptability, appropriateness, adoption, feasibility, fidelity, implementation cost, coverage and sustainability) were applied to frame and describe the effectiveness of the national rollout of the AC programme in South Africa.

**Results:**

We identified 32 eligible documents that were included for analysis. Our analysis showed that ACs were highly acceptable by patients and health stakeholders given the observed benefits, including decongestion of clinics, increased social support for patients and the low cost of implementation. Evidence suggests that the AC model proved to be effective in improving adherence to ART and retention in care. Based on the success of ACs in the Western Cape, ACs are currently being implemented in all of the other South African provinces.

**Conclusion:**

The inherent adaptability of the AC model should allow innovative strategies to maximise the use of existing resources. Therefore, the challenge is not limited to acquiring additional resources and support, but also includes the efficient use of available resources. Emerging challenges with AC programmes need to be addressed by increasing communication between stakeholders and fostering a culture of learning between facilities. As the AC programme expands and adapts to accommodate more people living with HIV and different population groups, policies should be designed to overcome present and anticipated challenges to enable its success.

## Introduction

Statistics from the latest South African national HIV survey showed that there were 7.9 million people living with HIV (PLHIV) in South Africa in 2017. Of these, approximately 62.3% were reported to have access to antiretroviral therapy (ART) in the same year [1]. Consequently, South Africa is considered to have the largest HIV-treatment programme in the world [2], accounting for approximately 20% of people on ART globally [3].

**Fig. 1:**
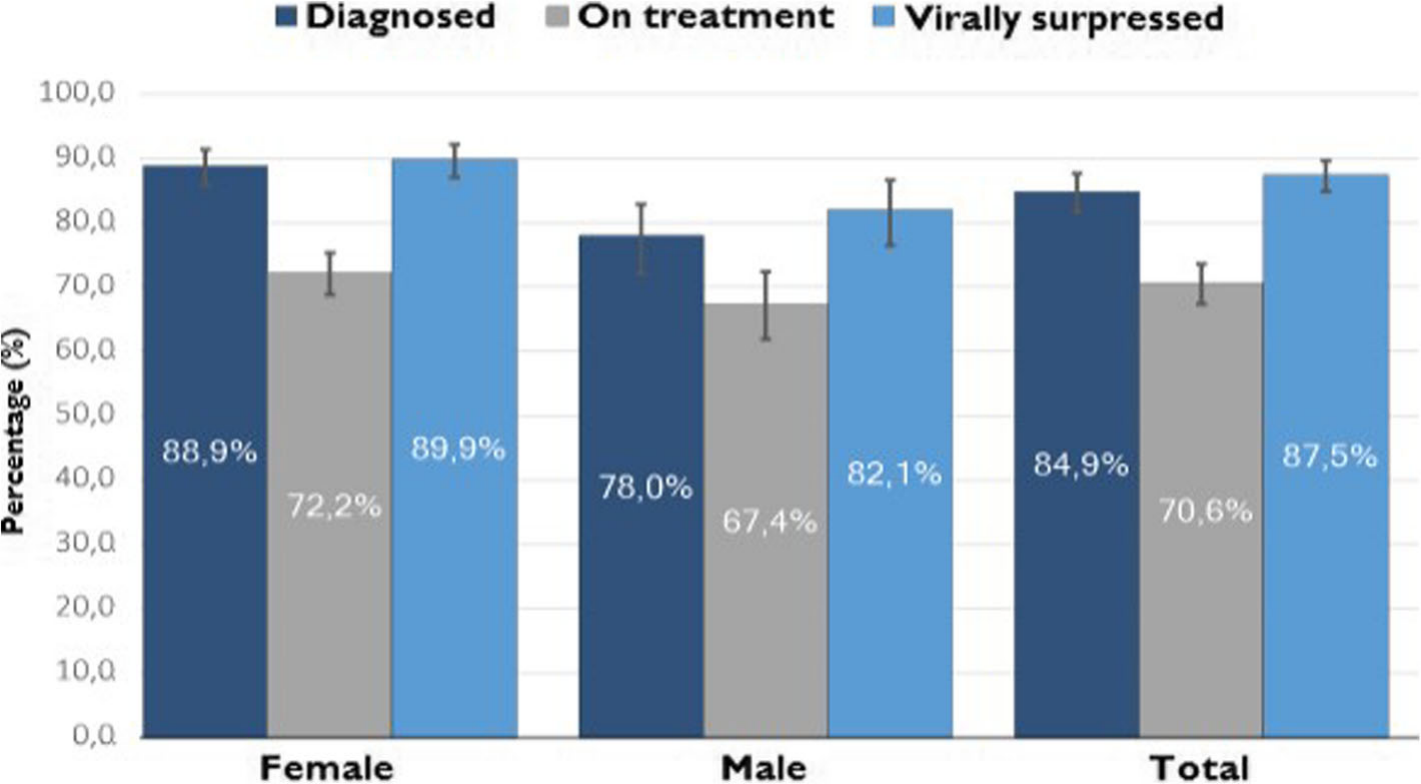
Viral load suppression among people living with HIV on antiretroviral treatment in 2017 [1]

In comparison to the UNAIDS’ 90–90–90 goal (90% of PLHIV diagnosed, 90% of those diagnosed linked to ART, and 90% of those on ART to achieve viral suppression) by 2020 [4], an estimated 86% of PLHIV were aware of their HIV status, 70.6% of those aware of their status were on treatment, and 87.3% of those on treatment had achieved suppressed viral loads in South Africa as of 2017 [1] (Fig. 1). The Human Sciences Research Council report also indicated that only 62.3% of all PLHIV in South Africa were virally suppressed, irrespective of treatment status [1].

As HIV programmes mature and increase in size over the years, the need to ensure long-term retention in care (RiC) of patients receiving ART while continuing timely initiation of new patients onto treatment presents an on-going challenge to healthcare providers and policy-makers [5]. Figure 1’s illustration suggests that retention in ART and population-level suppressive adherence to antiretroviral medication, although improving (almost achieving the 90–90–90 goal), remain critical issues to be addressed by the South African health system. The success of the rapid ART initiation in South Africa has put pressure on health services to deliver consistent quality care, including timely access to medication, follow-up of defaulters and monitoring of drug resistance [6]. To improve timely initiation to ART for naïve HIV patients while retaining those already in care, various differentiated ART delivery models were proposed [7].

Differentiated ART delivery models are ancillary to the mainstream ART delivery schemes, and they streamline ART service delivery by adapting the care components to the needs of the targeted group [8]. Common differentiated ART delivery models implemented in South Africa include facility- and community-based adherence clubs (CBAC), quick pharmacy pick-up and community-based pick-up. Of these proposed differentiated ART delivery models, adherence clubs (ACs), which were originally designed and implemented in the Western Cape (WC) Province by Médecins sans Frontières (MSF) [9], have shown better adoption prospects in South Africa.

## The AC programme

**Fig. 2:**
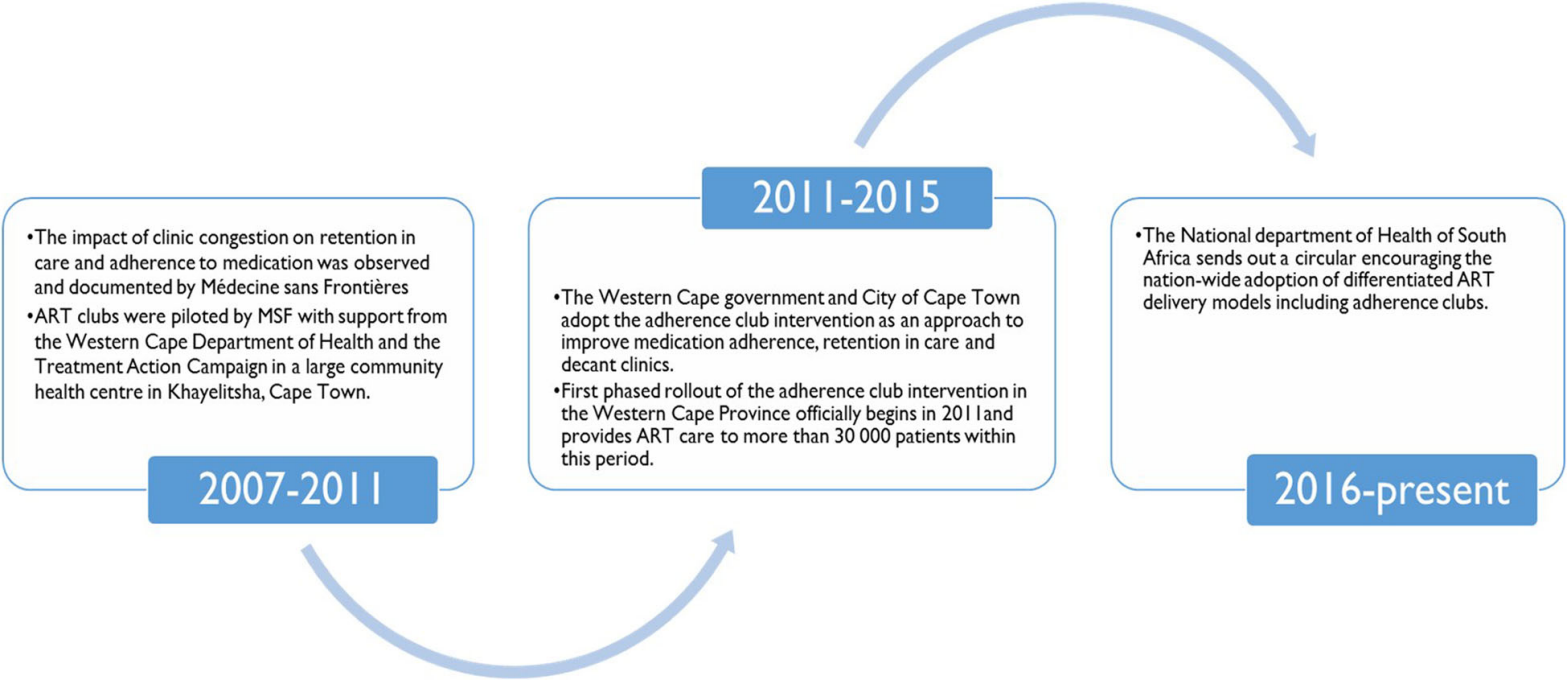
Important timelines in the development and adoption of the adherence club intervention in South Africa

The AC is a group-based adherence-enhancing intervention designed to address the challenges of clinic congestion, poor RiC and adherence to ART [10]. The AC intervention (1) retains patients in ART care by providing a more efficient way to manage stable patients; (2) achieves and maintains good long-term adherence in PLHIV on ART by maintaining good quality care and creating a convenient environment for adherence support visits that accommodates their lifestyle needs; and (3) decongests the health facility through group sessions that are facilitated by trained non-clinical staff [11]. Figure 2 illustrates the important timelines of the development and adoption of the AC intervention in South Africa. As the AC intervention evolved through the years various variations to its adoption have been noted. Table 1 outlines the components of an AC and some implementation variations.

**Table 1:** Adherence club (AC) model components and possible implementation variations

Components of the ACs	Possible implementation variation
Eligibility criteria	Duration of time on ART required (6–12 months)
	Inclusion of patients on second-line ART
	Number of suppressed viral loads required
	Inclusion of patients with comorbidities
	Retain pregnant patients
Location of AC meetings	Within ART facility
	Community venue close to the facility of patients’ homes
	Home of AC member
Cadre of staff facilitating the AC	Lay counsellor
	Community health worker
	Nurse (professional or auxiliary)
	Pharmacy assistant
	Club member
ART dispensing strategy	Pre-packed at central dispensing unit
	Pre-packed at health facility
Integrated services provided	Condom distribution
	Family planning
	Hypertension/diabetic drug supply
Patient population	General adult population
	Adolescents/youths
	Families

**Fig. 3:**
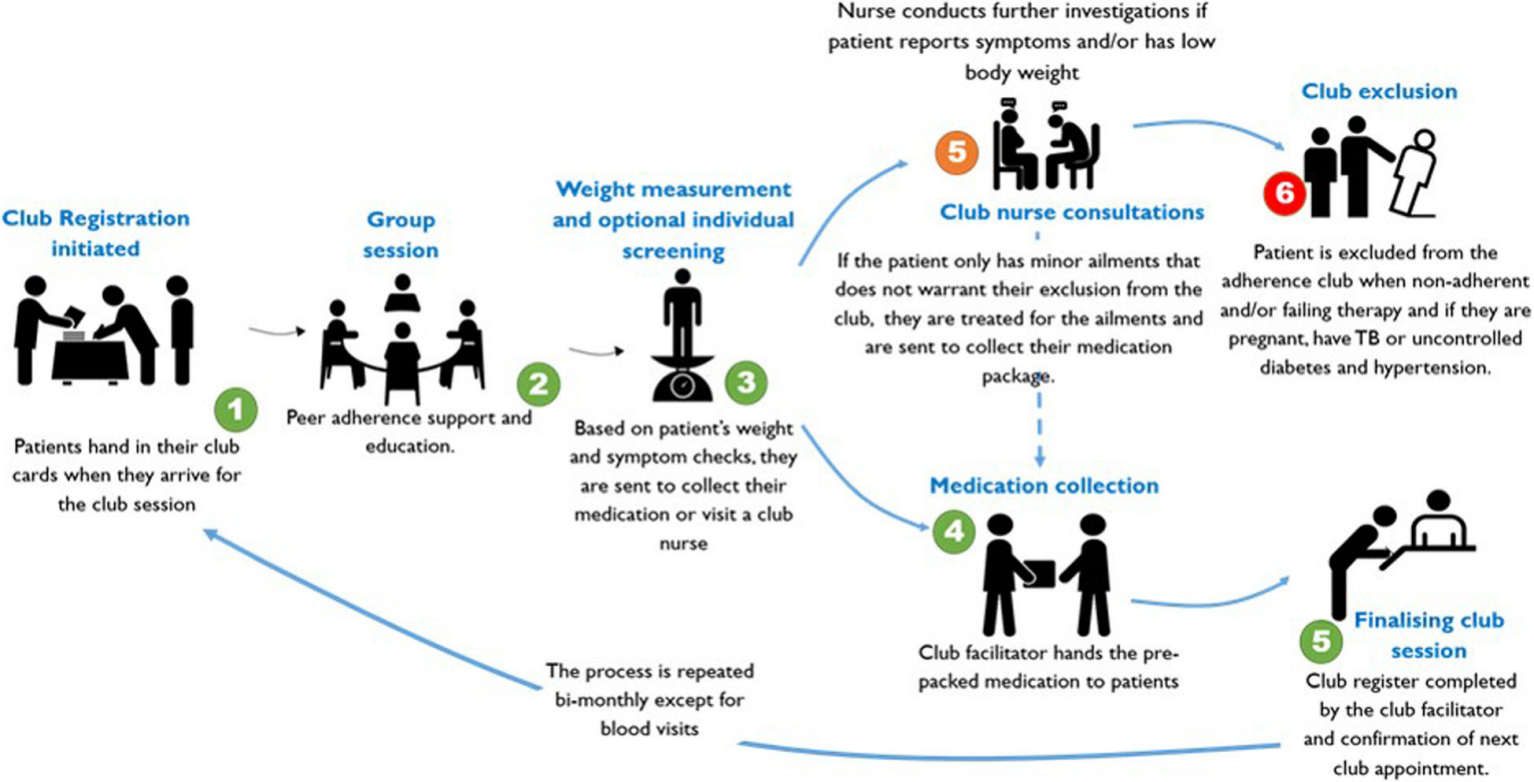
The operation of the adherence club intervention [12]

The activities of the AC are organised bi-monthly. A regular club session lasts approximately 1–1½ hours. On days when blood tests are carried out for routine adherence monitoring through CD4 count and viral load, the sessions take much longer. Once a year, club members attend regular clinical visits. A club patient can send a treatment ‘buddy’ to collect their medication within a ‘grace’ period of 5 working days. Figure 3 illustrates the AC main activies and processes.

Through group consultations, convenient medication pick-up processes and providing access to a clinician when needed, the AC model drastically reduces waiting times for patients. The intervention also provides a social environment that encourages patient interaction. Mukumbang et al. [12–15] found that by grouping clinically stable patients on ART in a convenient space to receive a quick and uninterrupted supply of medication, health talks, counselling and immediate access to a clinician when required, their self-efficacy improves and patients become motivated and nudged to remain in care and adhere to their medication.

Although empirical evidence demonstrates that the AC models are more effective in retaining patients in care and improving supressive adherence to medication compared to the regular clinic ART services [16–18], anecdoctal reports indicate that the rollout of ACs in South Africa to date is substandard. To this end, we aimed to explore and describe the intentional actions to adopt and rollout ACs nationally using the implementation outcome variables – acceptability, adoption, appropriateness, feasibility, fidelity, implementation cost, coverage and sustainability [19]. The implementation outcome variables serve as indicators of the successes and challenges of rolling out and implementing the AC intervention. To this end, we aim to assess what the implementation outcome variables indicate about the scaling-up of ART ACs in South Africa.

## Methods

### Study design

We conducted a qualitative document review analysis on AC implementation in South Africa to date [20]. Document reviews are used to gather background information and are particularly useful for determining whether a programme implementation reflects the original programme plans [21]. Therefore, this approach is aligned with the study aim as it guided us to produce rich descriptions of the AC intervention to provide evidence that explains the effectiveness of the rollout of the AC intervention in South Africa.

### Search strategy

Our search strategy was designed to be broad to minimise the possibility of missing relevant documents and included all types of descriptive, explanatory and evaluation evidence. The search was conducted from December 2017 to July 2018. We searched various databases (PubMed, Google search, and Google Scholar) and relevant websites; the National Department of Health (NDoH), MSF, WC Department of Health and Health E-news, using the terms “adherence club”, “ART adherence club”, “ART clubs”, “facility-based adherence club”, “MSF innovation in ART management”, “differentiated ART care model”, and “differentiated ART delivery model”.

### Data analysis

We followed a thematic framework synthesis approach [22] by adopting the implementation outcome variables described by Peters et al. [19], namely acceptability, adoption, appropriateness, feasibility, fidelity, implementation cost, coverage and sustainability. The implementation outcome variables assess how well implementation has occurred regarding the intentional actions to deliver health services [19]. The implementation outcome variables were selected over other existing implementation frameworks such as RE-AIM and PRECEDE-PROCEED because they encompass the full range of concepts now thought to be involved in the implementation of innovations in healthcare. Bennet et al. [23] demonstrated the role and feasibility of using the implementation outcome variables when they employed them to conceptualise information from reviewed project documents from three countries to address questions regarding the scalability and sustainability of innovations.

FCM and ZO coded the documents included in the review independently according to the implementation outcome variables. In a discursive process, the authors deliberated on the appropriateness of the codes to classify identified themes within the various implementation outcome variables [24]. The discursive process was iterative – moving from the codes through the identified themes and implementation outcome variables – as some of the implementation outcome variables overlap. We repeated this exercise until the evidence could be presented in a table appropriately matching the framework [22].

## Results

### Nature of selected documents

Table 2 illustrates the type of documents included in the review and the frequency of each type of document.

**Table 2:** Summary of articles included in the document review (*n* = 32)

Type of document	Number of documents	References
Journal article (research)	13	[5, 13–17, 25, 26, 28, 31, 42, 53, 54]
Journal article (perspective)	4	[9, 32, 40, 45]
Website article	8	[27, 29, 30, 33, 35, 46, 51, 55]
Thesis	1	[34]
Conference presentation	4	[36, 37, 41, 43]
Process evaluation (NGO reports)	2	[38, 39]
Total	32	

### Thematic reporting based on the implementation outcome variables

Thirty-two documents were reviewed and coded according to the themes of acceptability, adoption, appropriateness, feasibility, fidelity, implementation cost, coverage and sustainability, to reflect the state of the AC rollout in South Africa (Table 3).

**Table 3:** Thematic reporting based on the implementation outcome variables

Implementation outcome	Applied definition	Quotes from various sources
Acceptability	The perception among stakeholders that an intervention is agreeable	"But the contrast between her experiences at the clinic and the adherence club is more than just a matter of efficiency, she said. At club meetings, there’s a strong sense of solidarity, reassurance and openness" [53] "The adherence club intervention also offers cost and time benefits to the health system" [5] "The purpose of the adherence clubs from the healthcare worker’s point of view was to streamline clinic functioning and create conditions for faster processing of already-adherent patients" [25]
Adoption	The intention, initial decision or action to try to employ the new intervention	"The Global Fund to Fight AIDS, TB and Malaria, the country is planning to move treatment out of clinics and into patients’ homes, local libraries and maybe even their local chain-clothing store, like PEP, in the next three years" [54] "Expansion of the model to include groups of relatively healthy HIV-infected individuals with inequitable access to and outcomes on ART, such as women who initiate ART in pregnancy, should be considered" [42]
Appropriateness	The perceived fit or relevance of the intervention in a particular setting or for a particular target audience (for example, provider or consumer) or problem	"The ART-adherence club model improves adherence and long-term retention in care among clinically stable ART patients while optimising health resources to manage new ART patients and patients at risk of failing treatment" [9] "We observed an increased risk of loss to follow-up and viral rebound in younger patients; a finding that has been demonstrated in other studies" [18] "ART clubs were generally felt to be efficient, accessible, convenient, patient-friendly, supportive, accommodating of the needs of working people, as well as decreasing the workload of the ART clinic and hospital pharmacy" [26]
Feasibility	The extent to which an intervention can be carried out in a particular setting or organisation	"The AC represents ‘the model to follow in task shifting and task sharing to more community-based care’. But to scale up the model, South Africa must train hundreds of thousands of community health workers" [28] "Club availability has since been expanded and extended to all clinics in the sub-district, enabling ongoing increases in enrolment without additional clinical staff" [16] "A total of 129,058 HIV stable patients decanted from 330 clinics to 4971 community-based adherence clubs across 15 districts" [31]
Fidelity	The degree to which an intervention was implemented as it was designed in an original protocol, plan or policy	"Groups are now meeting in the community, introducing challenges in the delivery of pre-packaged drugs to community settings, and the collection, transfer and review of patient and programme management data" [16] "Fifteen locals, each carrying a small, green medication booklet, gather in the home of a woman who’s hosting a meeting of an adherence club for HIV-positive people" [40] "The club model grew out of these experiences but appeared to have diverged somewhat from the earlier ‘support group’ ideas and dimensions of adherence counselling and peer support, focusing more on convenience for those with HIV" [32] "The focus is on people taking ART who are clinically stable and includes those on ART who also have non-communicable diseases such as hypertension and diabetes" [45]
Implementation to cost	The incremental cost of the implementation strategy and the cost of the intervention itself	"Group-based models of chronic care are patient- friendly, budget-friendly and efficient ways of delivering ART in areas with a high burden of disease" [26] "Saves the health system and patients invaluable time and money" [35] "All costs were lower in the clubs compared to standard of care" [46]
Coverage	The degree to which the eligible population actually benefits from the intervention	"AC were implemented across the Cape Metro health district over a 4-year period providing ART care and support to more than 30,000 patients" [9] "We are setting up over 150 clubs across the provinces of Mpumalanga, Gauteng and the Free State at the Department of Health facilities" [45] "Adherence clubs in KwaZulu-Natal, the Eastern Cape and the Western Cape are also being rolled out" [45] "AC club models were implemented in 10 facilities in Tswane, 10 facilities in Nkangala and 15 facilities in Capricorn. From July 2016 to December 2016, 1500 patients were enrolled in Tswane, 850 in Nkangala and 5524 in Capricorn, with retention rates of 90%, 94% and 95%, respectively" [51] "MSF’s HIV adherence clubs have grown, in under two years, to include 11,000 members in the Mangaung district" [55]
Sustainability	The extent to which an intervention is maintained or institutionalised in a given setting	"There is a need to establish a working relationship with the health facilities that will lead to adoption and validation of the adherence club registers used in communities" [33] "As a lot of the work of running the adherence clubs is administrative in nature, staff members were in agreement that ways had to be found to streamline work and involve non-clinical staff so that clinicians could be freed up to schedule regular check-ups and attend to sick patients" [26] "The management of these groups is becoming increasingly challenging as the number of groups associated with a single clinic increases, requiring new management strategies and related research" [16]

## Discussion

The results from the document review provide evidence for the national rollout of AC in South Africa. Using the implementation outcome variables, we provide insights into how well implementation has occurred regarding the intentional actions to rolling out the AC intervention in South Africa.

### Acceptability

Based on the evidence reviewed, ACs demonstrated high acceptability among patients and healthcare workers (HCW). Patients attending ACs were generally enthusiastic about the innovation, citing both psychosocial and structural benefits as reasons for their acceptability [14, 17, 25–27]. ACs provide patients with a space to form cohesive and positive group dynamics over time, thereby rendering necessary peer support, which contributes to adherence to ART [9, 28]. Pre-packaged medication dispensed at the clubs proved to be more efficient and convenient for working patients, who no longer need to miss days of work to attend the clinic [25, 26]. Evidence suggests that the reduction of clinical visits and ARV pick-ups may improve clinical outcomes of AC members [29].

As highlighted by Grimsrud et al. [30], the current ART pharmacy guidelines in South Africa require ART to be scripted every 6 months. Although national policy allows 3-month dispensing, there is great variation between provinces and individual facilities. In the standard AC medication pickup, patients receive a maximum of 2 months’ supply. Nevertheless, to support ART patients who most commonly migrate over the Christmas holiday period, most clinics have resorted to providing 4 month’s ART supply, dispensed as two 2-monthly supplies to align with the national policy [30]. Regrettably, data are limited on how long ART dispensing intervals should be to optimise RiC. A comparison of outcomes among AC members who received 2-month ART (normal standard of care) to 4-month ART showed no difference in defaulting or viral suppression between both groups of patients [30].

HCWs are in agreement concerning the benefits of ACs for patients and the healthcare system. The implementation of AC serves to decongest facilities and streamline ART delivery, thereby ensuring that stable patients move through the ART clinics at a faster rate. Consequently, HCWs have more time to devote to new and ill patients, thereby improving infection control and providing health promotion opportunities [25, 26]. Additionally, HCWs are in favour of moving ACs from health facilities to community settings to reduce distance barriers to medication access, promote patient empowerment and enhance self-management while relieving clinic congestion [27, 31–33].

While the benefits of the AC prove to be acceptable to both HCWs and patients, the management of the clubs is not without challenges. During the pilot phase, the packaging of ART was done by the respective facility pharmacies. However, responsibility has since been taken over by the chronic dispensing unit (CDU), which is operated by a private logistics company on behalf of the WC Department of Health. Various studies have shown that the CDU often fails to deliver medication to the clinics on time, and in some cases, the delivery included incomplete packages [25, 26, 32, 34]. This problem is more bureaucratic in nature and reflects a lack of communication between the HCWs and the CDU [25]. A setup similar to the CDU in the WC, the centralised chronic medicines dispensing and distribution, has been rollout in the National Health Insurance districts in other provinces since 2014.

Although the conceptualisation of ACs to be run by trained lay counsellors relieves clinicians to focus on unstable patients on ART [7, 21], the operation of ACs requires a great deal of administrative work [16, 32] and some degree of support from clinical staff. This contradicts the goal of the clubs, as the clinical staff is required to be involved in administrative duties rather than clinical care. There is a need to streamline work and involve more non-clinical staff to take over logistical duties [25, 26]. According to Macgregor et al. [32], the small-scale AC implementation showed promising outcomes and received the necessary support regarding the supply of human resources, particularly trained club facilitators. However, the rapid scale up of the clubs sees the human resource support diminish.

### Adoption

The MSF pilot of ACs in Khayelitsha demonstrated promising results of ACs improving ART adherence and RiC [35, 36]. This led to the expansion and scale-up of ACs across the Cape Metro health district between January 2011 and March 2015 [9, 36]. In 2016, the South African NDoH recommended the use of differentiated ART delivery models nationwide to decongest the healthcare facilities and encourage adherence to medication by PLHIV on treatment [37]. NGOs such as the National Religious Association for Social Development (NRASD) and Care and Support for Improved Patient Outcomes (CaSIPO) have received funding to rollout ACs across South Africa following the NDoH implementation guidelines [38, 39].

According to Champion [40], the AC model is being rolled out in South Africa because it provides a cost-effective strategy for improving long-term retention and compliance with ART. During the expansion phase, workshops were conducted with various stakeholders, including MSF, Witkoppen, PEPFAR Liason and South African partners, for the purpose of providing information and updates concerning the AC expansion and rollout [34, 41]. According to Wilkinson [9], the goal is to further develop the AC models to reach full decentralisation of AC into communities.

### Appropriateness

Our document review indicates that the AC intervention is effective (appropriate) in improving RiC, adherence to medication and decongesting healthcare facilities. According to Wilkinson [9], the MSF pilot project reported that retention in clinic care after 40 months was 97% for club patients compared with 85% among those who qualified for clubs but continued to be managed outside of the club model. Other studies also reported that AC attendance was associated with a significant decrease in the risk of loss to follow-up (LTFU) and virologic rebound compared to patients receiving facility-based ART care [9, 16–18, 42].

Studies have indicated, however, that younger people continue to be at risk for LTFU and virologic rebound, despite their participation in the clubs [18, 42]. A study conducted by Grimsrud et al. [42] demonstrated that youths aged 16–24 years were the only group not to show significant improvements in LTFU and virologic suppression when comparing CBAC to standard care. According to Tsondai et al. [18], young people face specific challenges in managing chronic health issues, which may adversely impact their adherence to ART and RiC. This suggests that, while ACs in their current form could be appropriate for the management of ‘stable’ adult patients on ART, they may not appropriately meet the need of adolescents and young people on ART. Therefore, ACs should be designed to address the specific needs and expectations of adolescents and young adults [18, 43]. A recent study conducted by MacKenzie et al. [38] showed that adolescent-centred teen clubs retained more adolescents in care compared to standard clinic care; suggesting that teen clubs may be effective in reducing attrition from ART among adolescents [38].

While facility-based and CBAC models provide adherence support to patients with regard to adherence to medication and RiC, CBACs contribute more substantially to the decongestion of health facilities, with patients only required to attend the facility for annual laboratory tests and clinical consultations [31].

### Feasibility

According to Wilkinson [9], the acceptability and feasibility of the AC implementation are reflected in the pace of scale-up, the proportion of patients attending the clubs, and the size of the AC programme. Over the 4-year period of rapid scale-up, 25% of the Cape Metro health districts’ ART cohort was shifted to receive ART in the AC model. According to Wilkinson [9], each facility running an AC requires a club team consisting of a part-time club manager, at least one full-time lay facilitator per 40 ACs, and a club nurse. To meet the needs of the AC staff, the Cape Town Metro has included club facilitation in the job profile of facility counsellors, and additional counsellor posts have been allocated to facilities – one for facilities with more than 15 clubs, and two for facilities with more than 40 clubs [9].

The advantage of the AC model, which increases its feasibility, is that it allows for adaptions to contexts based on existing resources. For example, AC meetings can be held in members’ homes or accessible community venues, and resources can be saved by adapting the AC schedule from 2- to 3-monthly meetings [9, 17, 28, 40]. The expansion of club programmes and the appropriateness of the intervention demonstrate that it is possible to implement clubs within the current health systems context. However, the increase in club numbers will require more human and structural resources to support their continued development [28, 32, 36].

After 2 years of experience in establishing CBACs across 15 districts in eight provinces, the CaSIPO Project [39] identified the use of ward-based primary healthcare outreach teams and NDoH-funded community-based organisations as cost-effective, scalable and sustainable models. Furthermore, the use of community health workers (CHWs) from funded community-based organisations for the facilitation of the ACs strengthens the linkages with the decanting clinics and facilitates a two-way referral pathway [31].

### Fidelity

ACs have certain core components for the purpose of providing a structured programme of implementation. However, even during the 2011–2015 expansion phase, variations in the implementation of the model were identified [36]. A process evaluation commissioned by the NRASD reported that the different regions vary in their ways of implementing AC; with some facilities showing more leniency regarding the recruitment criteria [44]. Flexibilities within the AC model allow for adoptions to local contexts, thereby ensuring optimal performance [17].

Initially, patients were eligible to join an AC if they were ‘stable’, being on ART for at least 18 months, had two consecutive suppressed viral loads, a CD4 cell count above 200 copies/mL, the absence of other chronic conditions and a referral from a clinician [16, 36]. The eligibility criteria were amended in 2013 and then again in 2015, by discarding the CD4 cell count criterion, reducing time on ART treatment to 6 months and one viral load suppression [36]. However, in practice, more considerations are taken into account when it comes to allowing patients into ACs.

More recently, the eligibility criteria for admission into the AC have been amended to include patients who are clinically stable but have comorbid non-communicable diseases such as diabetes and hypertension [44, 45]. In other words, if a patient in the club develops a chronic comorbidity that is well-controlled and if the patient is ‘stable’ as per the clinician’s assessment, then they will still be eligible for a club on condition that they fulfil ‘HIV-related’ criteria [12]. In a similar manner, some facilities choose to keep pregnant women in the club as long as their viral load is undetectable, in which case their antenatal visits are managed separately by the staff responsible for antenatal care [12]. However, in some settings, pregnant women are placed out of clubs and are only allowed to re-enter after their pregnancy, particularly when mother and baby will be managed as a pair in the post-natal period or if antenatal care is delivered at a facility other than that rendering club services. Changes in eligibility criteria allowed for more ‘stable’ patients to be included in the club, thereby freeing up more space in the ART clinics for newer patients.

Since its inception, the AC model has evolved in various ways. The evidence shows that the goal of decentralising ACs into communities is being realised [9, 17, 33]. The CBACs are facilitated by a CHW at a community venue or a home within the community [28]. The CBACs operate in a similar manner to the facility-based clubs, with the benefit of being more accessible to patients by reducing transportation costs. Furthermore, there has been a move towards establishing AC developed specifically for young people or men, as these groups are most difficult to retain in care [40]. The variations and changes in the implementation of the AC model since the expansion phase are aimed at improving the effectiveness of the model.

### Implementation to cost

The evidence of the document review demonstrates that the AC model is cost-effective compared to standard care [9, 12, 35, 46]. The cost-effectiveness of the model is attributed to the use of a CDU to deliver pre-packed medicine to facilities, thereby reducing pharmacy-related bottlenecks and congestion in facilities [9, 25, 28, 46]. Furthermore, group-based models of chronic care have been proven to be more budget-friendly to patients and facilities [26]. According to Bango et al. [46], the use of lay health workers in community-based care is more cost effective than facility-based care. As previously mentioned, the AC can be adapted to local contexts to ensure that the model fits in with existing resources. However, the scale-up of the AC requires sufficient human resources to support and run the clubs [35].

While the NDoH provides funding [31] for the AC implementation, the initial success of the AC attracted the attention of various donors who saw value in funding the national rollout. NGOs such as NSRAD and CaSIPO have received funds from the Global Trusts [44] and USAID [31], respectively, to support the continued rollout of the AC. There are, however, concerns around sustained and long-term funding of the AC innovation.

### Coverage

#### Population

The pilot project was designed to include ‘stable’ adult (18 years plus) patients. Therefore, patients younger than 18 years and pregnant women were initially excluded from the programme. A study by Myer et al. [47] provides tentative evidence relating to the usefulness of AC for post-partum women initiating ART. According to Odendal [48], the MSF supports the implementation of family ART clubs, which focus on the long-term RiC of children stable on ART and their caregivers. While some facilities are running teen clubs, more research is needed to investigate possible models of AC for younger patients and post-partum women [18, 47].

#### Geographical

According to Wilkinson [36], more than 30,000 patients are receiving ART through the 2011–2015 AC expansion in the WC Province. According to Odendal [48], there are more than 400 established CBACs in the Cape Metro District. Since the inception of the AC intervention in the Cape Metropole of the WC Province, the AC programme has increased its coverage to other South African provinces, including Gauteng, Limpopo, Mpumalanga, Eastern Cape, KwaZulu-Natal and the Free State [45, 49, 50]. According to Skidmore [51], ACs have been implemented in 10 facilities in Tswane (Gauteng), 10 facilities in Nkangala (Mpumalanga) and 15 facilities in Capricorn districts (Limpopo) of South Africa.

The NRASD has implemented CBAC in the following regions: Free State (Lejweleputswa District, Thabo Mofutsanyane District), Gauteng (Sedibeng District), Limpopo (Mopani District, Sekhukhune District), Mpumalanga (Ehlanzeni District, Gert Sibande District), and North West (Dr Kenneth Kaunda District). Additionally, the Mosamaria project in the Free State [52] currently has over 14,000 members in their HIV Literacy and ART AC programme, which they aim to increase to 42,000 members by March 2019 [52]. The CaSIPO programme has established 5931 ACs across 15 districts in 8 provinces [31].

### Sustainability

The initial rollout of the AC has demonstrated some success. However, sustaining the clubs at a larger scale will be difficult if the emerging challenges are not adequately addressed. According to Macgregor et al. [32], acheiving large-scale AC implementation in the health system should be a continuous process. While the ACs have proven to be cost-effective, rapid scaling-up will require further resources to support club maintenance. Emerging logistical challenges already highlight the need to increase non-clinical staff and CHWs.

While CBACs show promising results, there is evidence to suggest these clubs present with increased logistical challenges [17, 34]. CBACs are faced with challenges inherent to their location outside of the facility, including having a qualified facilitator, ensuring the ongoing supervision of the clubs, finding a suitable venue in the community and transporting the patients’ pre-packed medicines to be distributed during the club sessions. Within South Africa’s diverse landscape, the establishment and maintenance of CBACs need to adapt to the local context and use innovative approaches to overcome these challenges.

Furthermore, there is a need to develop outputs documenting the practical implementation of clubs, and to distinguish between club rollout and scale-up [32, 34]. A particular concern threatening the sustainability of the clubs is the lack of communication and the systematic sharing of information across provincial health systems [25, 44]. To sustain ACs, there is a need to foster a culture of iterative learning to address emerging challenges and mitigate complex system errors and to establish a working relationship among all stakeholders [32, 33].

The quality of the services provided during the club sessions plays a critical role in the retention of patients in ACs. The skills and knowledge of the CHWs facilitating AC have a direct impact on the quality of the clubs and, in consequence, patient retention. CaSIPO used a mixed approach (training, mentorship and intensified technical assistance) to develop AC facilitation skills including nutritional assessment, counselling and support, and screening for TB and sexually transmitted infections.

## Study limitations

It is possible that our search strategy did not identify all the documents that could have been included in the review. Further, since the AC intervention was originally designed, piloted and rolled out in the WC province, most of the documentation and publications are understandably from this region of the province, which has the potential to introduce information or evidence bias.

## Policy implications and recommendations

The inherent adaptability of the AC model should allow for innovative strategies to conserve existing resources. Therefore, the challenge is not limited to acquiring additional resources and support, but also to the effective use of available resources. Emerging challenges within the ACs need to be addressed by increasing communication between stakeholders and fostering a culture of learning between facilities.

Appropriate grouping of patients in ACs is essential to ensure the full benefits of the clubs. Quarterly cohorting of patients according to their ART start date facilitates the management of the clubs as all patients from the ACs are due for their yearly clinical blood tests and clinical examination at the same time. In addition, supervision and monitoring of the facilitators promote quality in AC facilitation and record keeping (AC registers).

Potential legal restrictions to the rollout of ACs outside the healthcare facilities have been highlighted, especially regarding medication dispensing, as it is legally required for a nurse or medical staff to transport medicines outside the healthcare facilities. To overcome this challenge, according to MacGregor et al. [32], a new cadre of low-level pharmacy workers has also been approved to address legal grey areas in terms of dispensing ART off-site for the community- and home-based AC models. Policies enhancing the sustainability of this level of pharmacy workers would enhance the sustainability of the community- and home-based ACs.

Following the two 2-monthly (4 months) ART supply provided to ART patients gaining traction, and with evidence indicating that longer ART supply refill intervals over holiday periods does not have a negative impact on patient outcomes [30], some considerations have been made with regard to establishing clubs with 3 and 4 month medication supplies to further reduce the number of club attendees. Nevertheless, the change from the original 2 months’ supply is not evident at policy level [32].

Although an estimated 30 patients are required per club [9], there is evidence that some clubs could harbour more than 40 members [15]. We found a dearth of information with regard to how the number of patients per club influences the rollout, organisation and logistics of clubs per health centre. At the initial stages of the rollout of ACs, the systematic criteria for the identification of ‘stable patients’ for placement into clubs as established by MSF was crucial. Nevertheless, as the scaling up and diffusion of the intervention progressed, the entry and number of members per club criteria were altered to enable rising recruitment targets [32]. According to MacGregor et al. [32], albeit there being systematised procedures for starting clubs, there had been less effort to formalise plans for addressing the organisational complexity and challenges that come with a large increase in the number of clubs in a facility. Each healthcare facility seems to manage their own situation depending on the resources available to them. We propose that having clear policy statements and guidelines on how to deal with organisational complexities regarding the growing number of patients in clubs could be useful in the context of ‘test and treat’.

## Conclusion

The AC programme has demonstrated potential to improve RiC, enhance adherence to ART and decongest healthcare facilities offering ART. Evidence from the review suggests that the adoption of the AC programmes is appropriate and acceptable within the South African context as it is cost-effective and maximises the use of human resources. Its widespread adoption in the WC province suggests that it is feasible for a nation-wide rollout. The evidence suggests that other provinces have started implementing ACs with the promise of further expansion. However, rapid AC scale-up will require increased support at the policy level to ensure the model maintains its efficacy and sustainability.

## Abbreviations

AC: adherence club
ART: antiretroviral treatment
CaSIPO: care and support for improved patient outcomes
CBAC: community-based adherence club
CDU: chronic dispensing unit
CHW: community health worker
HCW: healthcare worker
LTFU: loss to follow-up
MSF: Médecins sans Frontières
NDoH: National Department of Health
NRASD: National Religious Association for Social Development
PLHIV: people living with HIV
RiC: retention in care
WC: Western Cape
